# Fully automated antibody structure prediction using BIOVIA tools: Validation study

**DOI:** 10.1371/journal.pone.0177923

**Published:** 2017-05-18

**Authors:** Helen Kemmish, Marc Fasnacht, Lisa Yan

**Affiliations:** Dassault Systèmes Biovia Corp., San Diego, California, United States of America; University of Edinburgh, UNITED KINGDOM

## Abstract

We describe the methodology and results from our validation study of the fully automated antibody structure prediction tool available in the BIOVIA (formerly Accelrys) protein modeling suite. Extending our previous study, we have validated the automated approach using a larger and more diverse data set (157 unique antibody Fv domains versus 11 in the previous study). In the current study, we explore the effect of varying several parameter settings in order to better understand their influence on the resulting model quality. Specifically, we investigated the dependence on different methods of framework model construction, antibody numbering schemes (Chothia, IMGT, Honegger and Kabat), the influence of compatibility of loop templates using canonical type filtering, wider exploration of model solution space, and others. Our results show that our recently introduced Top5 framework modeling method results in a small but significant improvement in model quality whereas the effect of other parameters is not significant. Our analysis provides improved guidelines of best practices for using our protocol to build antibody structures. We also identify some limitations of the current computational model which will enhance proper evaluation of model quality by users and suggests possible future enhancements.

## Introduction

Recent advances and success in using antibodies in treating diseases, including cancer, inflammation and rheumatoid arthritis [1, 2], have created great interest in designing new antibody biologics. Building three-dimensional models from protein sequences is frequently an important step in the antibody design process, enabling researchers to study antibody properties such as stability, antigenicity, aggregation propensity, solubility, viscosity, and more. In addition, when used in combination with protein-protein docking methods, these models can be used to understand and predict antibody-antigen interactions.

Homology modeling is a well-established method, which has been shown to produce quite accurate models for a protein sequence if an X-ray structure of a protein with a sufficient degree of sequence similarity is available[3, 4]. The area of antibody design and engineering is a case for which homology modeling is particularly well suited, because in general the overall sequence and structural similarity between antibodies is very high. In particular, the framework regions of antibodies are very well conserved, with most of the variability occurring in the complementarity-determining regions (CDRs). This property of antibodies has led to the development of specialized structure prediction methods [5, 6, 7, 8, 9, 10, 11] which have been shown to outperform generalist methods [12].

Antibody structure prediction methods generally follow a two-stage approach. In the first stage, an accurate model of the framework regions (i.e. excluding the CDR regions) is constructed based on appropriate templates [5, 6, 7, 8, 9, 10, 11]. The framework templates are typically selected based on sequence similarity from a curated database of antibody structures. Models are then built either based on a single template for the whole structure [5, 7] or separate templates can be used for the VH and VL chains [5, 6, 10, 11]. In the latter case an additional step is required to determine the relative orientation of the chains [5, 6, 11].

In the second stage, the hypervariable loop regions of the structure are rebuilt. Five of the six CDR regions typically adopt a limited number of conformations [13] and can in most cases be accurately modeled by grafting the regions from an appropriate template [14]. However, a number of different approaches are possible for CDR template selection and loop grafting [5, 6, 7, 8, 9, 10, 11].

The H3 loop is more difficult to model because this region exhibits a much larger degree of variation in loop length and conformations adopted. For H3, ab initio loop modeling methods have been shown to increase accuracy compared to template based models in some cases [7, 8, 9].

A blind prediction experiment assessing various antibody structure prediction methods was performed in 2009[15]. The results of BIOVIA’s participation in this experiment generally validated our template-based modeling approach. However, it also identified some deficiencies in our modeling process. The lessons learned allowed us to improve our performance in the second instalment of the Antibody Modeling Assessment (AMA-II), which was executed in early 2013 (http://www.3dabmod.com) [5, 12].

Based on the further experience gained from this, we developed a fully automated antibody modeling protocol which can be run through the Discovery Studio [16] graphical user interface or in batch mode from the command line. This method is very fast, and can be run on multiple processors with coarse grain parallelization. In addition, the protocol can process combined heavy and light chain inputs, matching separate heavy and light chains by name, or performing permutations combining a set of heavy and light chains. These features make it an ideal solution for structure prediction of multiple sequences. On a standard desktop PC, a single Fv or Fab structure can be predicted in less than 6 minutes; using a server with 30 processors predictions for a set of over 150 Fv sequences can be completed within an hour.

The fully automated method was initially validated using the sequences from the AMA-II experiments; that study included a diverse set of sequences, but consisted of only eleven targets. We designed the current work to extend the validation of our methods to 157 antibody sequences for which structures are available, and to analyze the influence of several parameters to obtain better understanding of the effect on model quality. This analysis improves the recommendations we can offer when using our protocol to build antibody structures.

## Materials and methods

### Computational methodology

The automated structure prediction method consists of three stages: Framework template selection, framework model construction, and CDR refinement. These are run in succession without manual intervention after the specification of the initial run parameters.

#### Framework template selection

Templates for each target sequence are selected by aligning against sequences in the Discovery Studio antibody database using a Hidden Markov Model [17], and then identifying those with the highest sequence similarity and identity. By default CDR regions are excluded from consideration but the user may choose to include them. The five best templates are found for the whole Fv or Fab region, and also for each of the light and heavy chains.

#### Framework model construction

We evaluated the following framework model construction methods implemented in the software:

1. Single Template: This is the most straightforward approach, in which a model is built based on a single Fv/Fab framework template. This template, which contains both the light and the heavy chain regions, is selected by sequence similarity from a curated antibody database.
2. Chimeric: This method builds a model based on a chimeric template. This template is assembled from separate light and heavy chain templates. A third interface template, containing the whole Fv/Fab region, is used to determine the relative spatial orientation of the individual light and heavy templates. The templates are selected from the database by sequence similarity for the relevant regions. Note that the light or heavy templates can be identical to the corresponding domain of the interface template.
3. Top5: This approach builds a model by using up to five Fv/Fab framework templates simultaneously. The five templates which have the best sequence similarity to the target are identified in the database. However, any template with a similarity not within 10% of the best one are rejected so occasionally fewer than five templates may be used. The models are built based on a multiple sequence alignment of these templates to the target sequence. This is done using the capability of MODELER [18] to construct models based on multiple templates by simultaneously optimizing restraints from all of the templates. MODELER uses an additive distance restraint function that peaks at the equivalent distance between atoms in each template. The contribution for each template is weighted by local sequence similarity, as described in detail in the MODELER paper [18].

In each case, one or more models are built using MODELER, and the top model as ranked by the MODELER PDF Physical Energy is used for further refinement.

#### CDR refinement

The top-ranking framework structure can then have any or all of its CDR loops refined. The CDR loops are located using the IMGT [19], Chothia [13, 20, 21], Honegger[22] or Kabat [23] numbering schemes.

Loop templates are identified based on alignment of the target to sequences in the antibody database which have identical CDR loop lengths. The templates may be filtered to use those which have the correct Chothia canonical type if available; the canonical type definitions [21] are shown in S1 File. The templates are ranked with a BLOSUM62 similarity score of the CDR region plus the stem residues. There is an additional ranking which favors templates that have high scores for the other two CDR loops in the domain. This can be beneficial as the conformation of the three loops may be interdependent. The final ranking is by crystallographic resolution. MODELER is used to build one or more new CDR models while keeping the framework region intact.

### Validation dataset

Validation of the method requires predicting the structures of antibodies for which the structures have been experimentally determined, but which are not yet present in the template database. Therefore, the computations were performed using the templates present in the Discovery Studio 4.1 database, while the validation set was created by searching the Protein Data Bank (PDB) [24] for newer antibody structures. Sequences were retained regardless of their similarity to those in the database because real usage often involves predicting the structures of a highly similar series of sequences, which may have identical frameworks or loop regions. This yielded an initial set of 249 Fv target sequences. Any structure with missing residues within the light or heavy chain was excluded. The set was further pruned to 95% sequence identity, choosing representatives with more complete termini and/or having structures with better crystallographic resolution. This resulted in a validation set of 157 unique Fv sequences. These are listed in S1 Text.

While most of the structures were of good or reasonable crystallographic resolution below 3.0 Å, 16 were in the range from 3–5 Å and three had been determined by electron microscopy with ‘resolution’ above 13 Å. The deviations between the experimental and predicted models for the electron microscopy structures are as likely to be due to inaccuracies in the deposited structure as in the prediction, so they were excluded from the analysis, leaving 154 structures.

The organism classifications of this final set are 75 human, 68 mouse, 5 rabbit, 4 rhesus macaque and 2 chicken antibodies; note however that this includes engineered structures. 125 have kappa light chains and 29 have lambda light chains.

**Fig 1:**
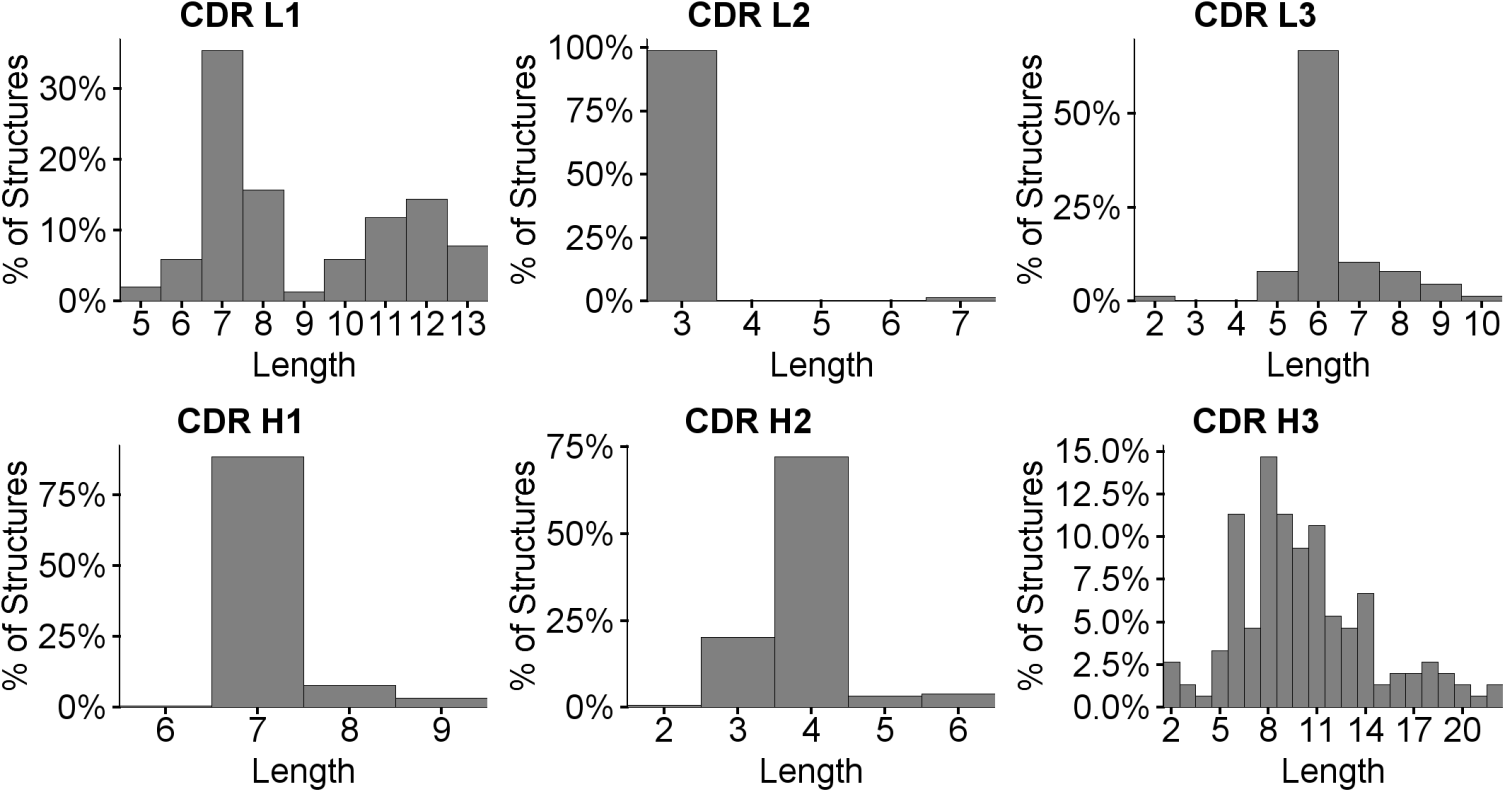
Length distributions for each of the CDR loops.

Loop length distributions, using the Chothia definitions, are shown in Fig 1.

**Fig 2:**
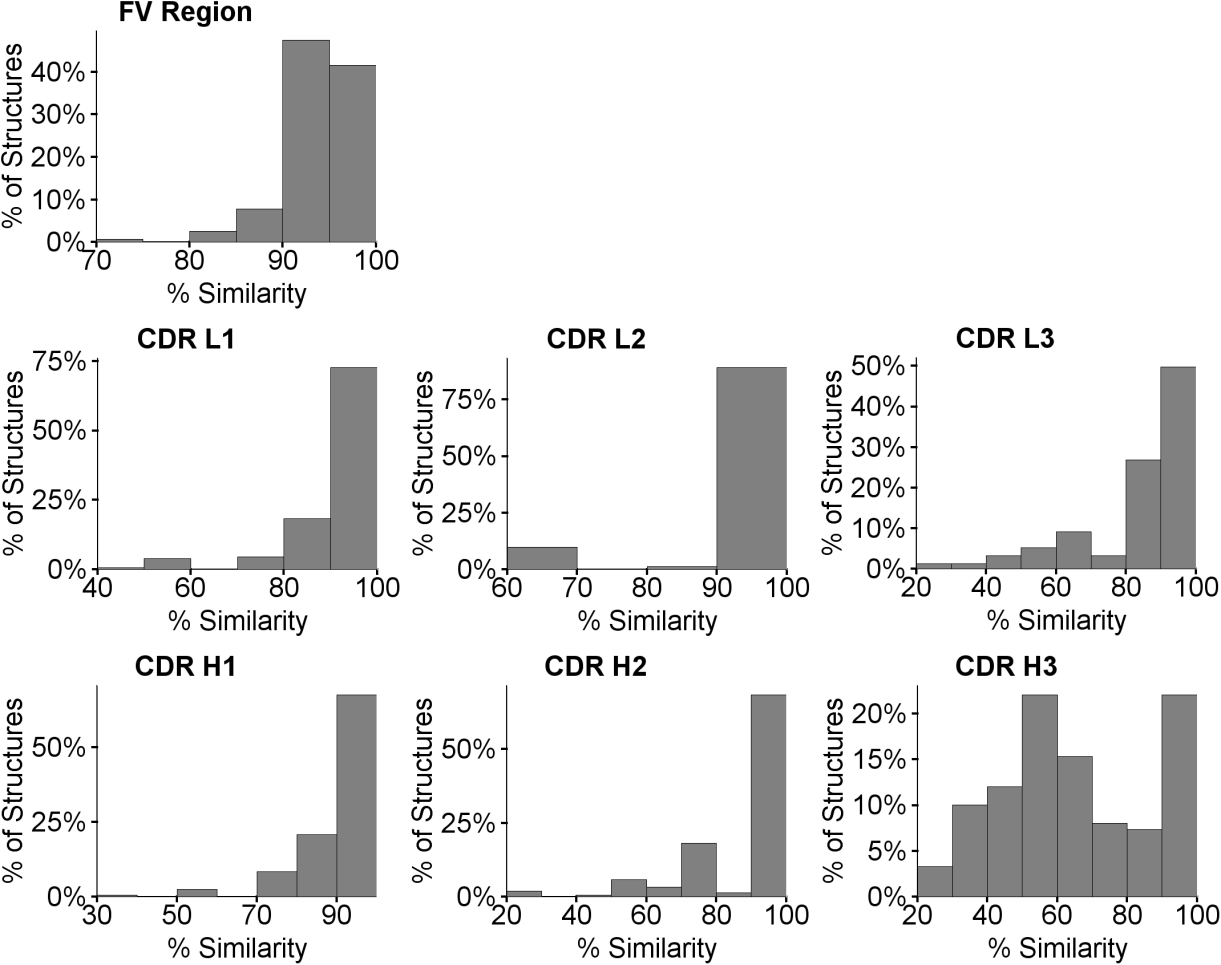
Distribution of sequence similarity of query sequence to database templates. CDR loops were defined using the Chothia numbering scheme.

The vast majority of the target sequences have at least one template in the Discovery Studio database with a sequence similarity above 90% for the Fv domain and above 80% for all CDR regions except for H3, as is shown by the histograms in Fig 2.

### Validation calculations

Structures were predicted for the validation set using a variety of the available options:

- Each of the framework template modeling methods
- Building different numbers of models
- IMGT, Kabat, Honegger and Chothia loop definitions, the latter with or without canonical filtering

#### Comparison of predicted and experimental models

The predicted models were compared with the experimental X-ray structures using the same methods as were applied in the AMA-II assessment [12]. This entailed superimposing each predicted-experimental pair using the β-sheet core, and then calculating RMSDs of the peptide carbonyl atoms for the light and heavy chain framework regions and for each of the CDR loops as defined by the Chothia scheme. Carbonyl RMSDs are used as they are sensitive to variations such as peptide flips which are not revealed by the commonly-used C-α RMSDs. In addition, the deviation in tilt angle between the light and heavy chain regions was calculated, again using the same method as for the AMA-II work [5]. The RMSD and tilt angle information, together with details of the templates used in each prediction, are tabulated in S1 Table.

#### Further analysis

Custom protocols were created in BIOVIA’s Pipeline Pilot to analyze and compare the predictions. Many of the results are presented using box plots which are a compact means of displaying the distributions for several sets of data on the same chart. The bottom and top of the box are at the first and third quartile respectively, while the line within it marks the median value and a dot marks the mean value. The ‘whiskers’ are calculated by the Tukey method as 1.5 of the lower and upper quartile ranges. In some plots, any outliers beyond these values are plotted as small squares, and this is the definition of ‘outlier’ used in parts of the discussion. The results obtained by different methods were also assessed using a pairwise t-test, with p values below 0.05 being considered to be statistically significant.

Detailed analysis was performed within Discovery Studio 4.5, utilizing its sequence alignment, structural superimposition and visualisation capabilities.

## Results and discussion

**Fig 3:**
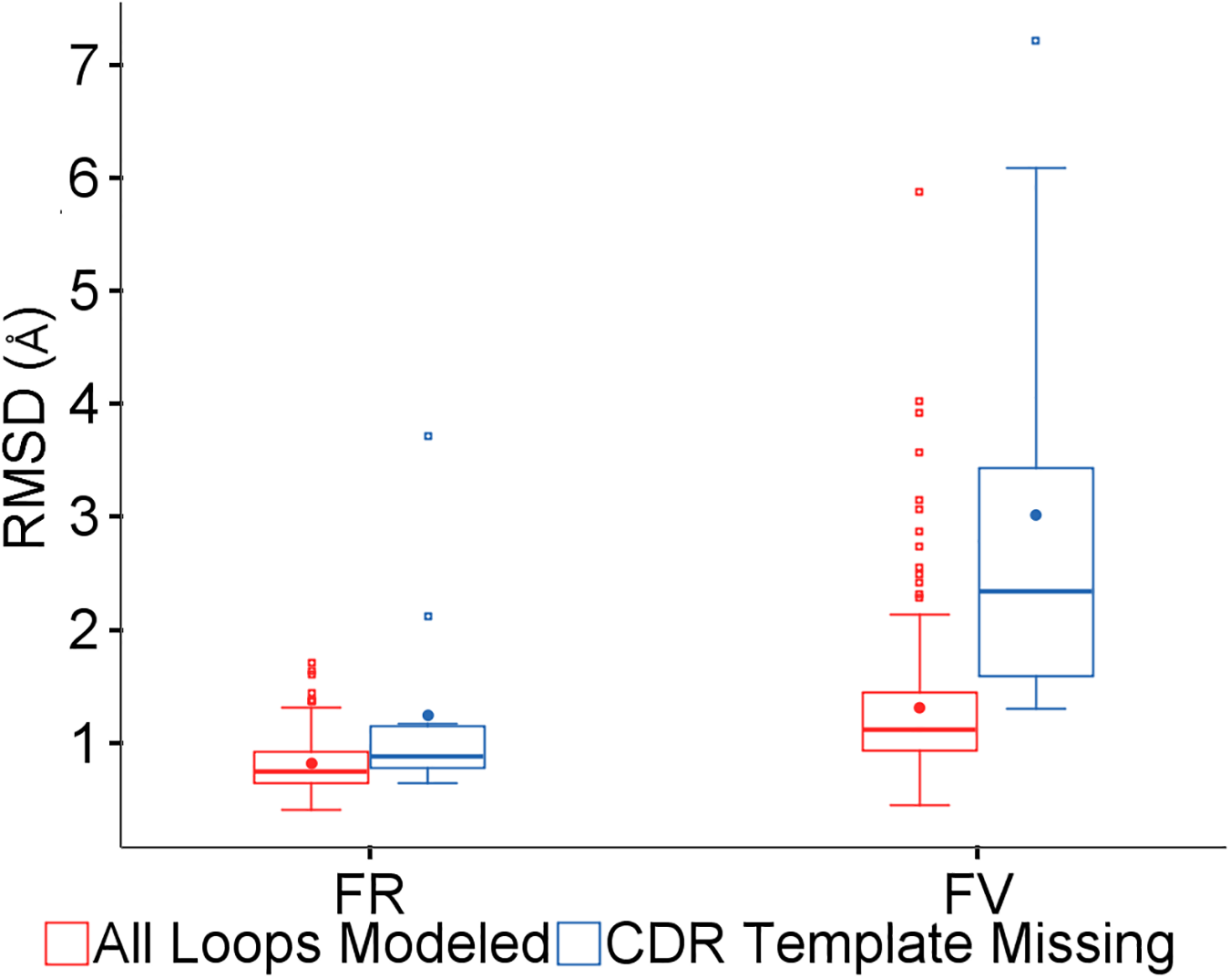
Effect of missing loop templates. Framework (FR) and overall (FV) RMSDs for predictions with all loops modeled or not.

Template based refinement of the CDR loops by homology modeling requires a template with identical loop length. The validation dataset contains 6 sequences in which no template was available for one of the loops using any of the loop definitions, and a further 4 and 5 cases respectively for the Kabat and Honegger definitions. Details can be found in S1 Text. The set of 11 predictions using the Honegger definition were examined; the similarities for the best framework model were all above 77%. The overall and framework RMSDs for this group versus the remainder of the set with framework similarity above 77% were compared. Fig 3 shows that in addition to the unsurprising decrease in accuracy of the overall RMSD, the quality of the framework models is also generally poorer. There are particularly large distortions if long CDRH3 loops are misplaced. These cases have been excluded from the remaining analyses, leaving 148 sequences for the Chothia and IMGT methods.

### Framework models

**Fig 4:**
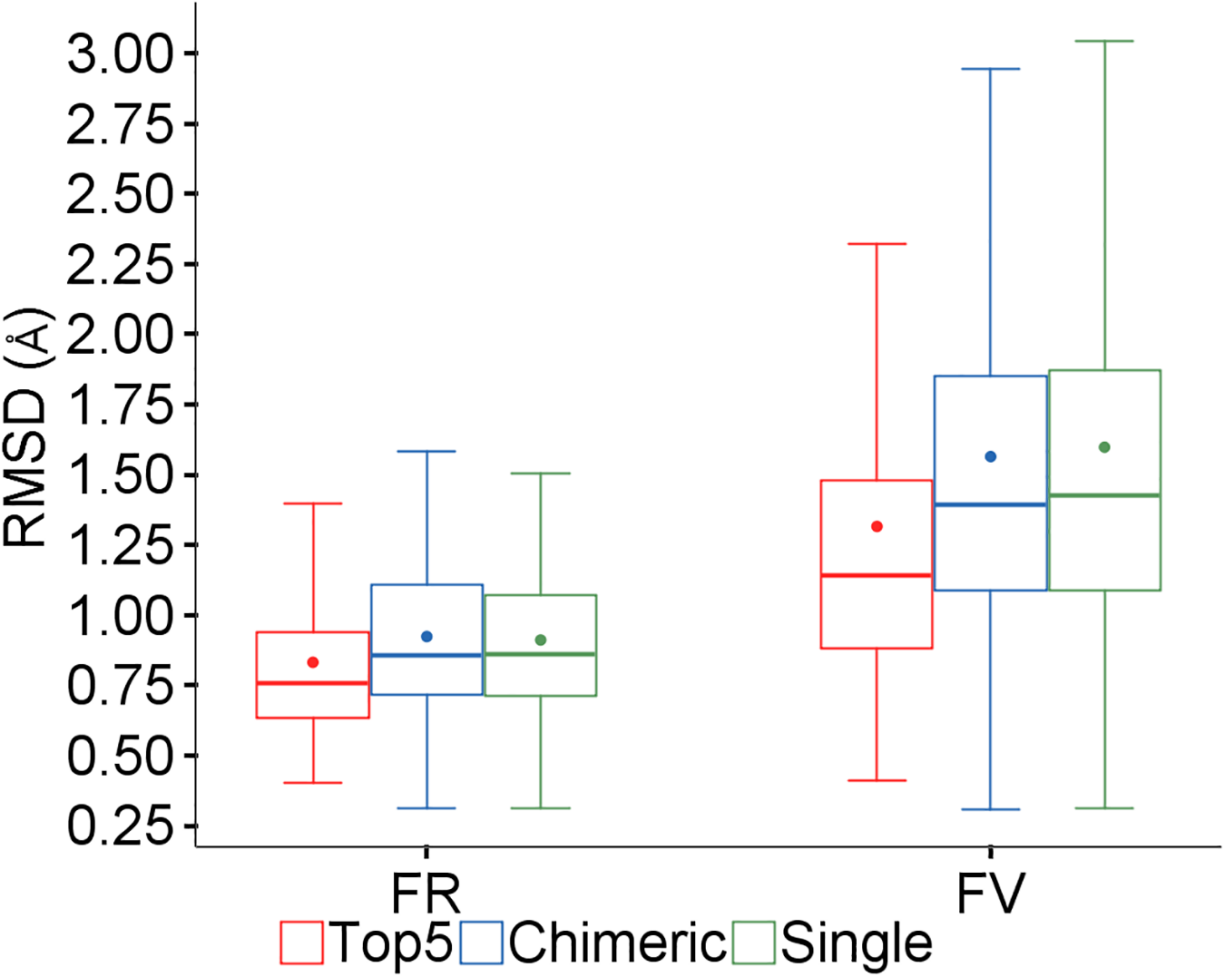
Comparison of framework modeling methods. Framework (FR) and overall (FV) RMSDs (Å) for the three methods.

The predictions were run for the sequence set using each of the three framework modeling methods (Single, Chimeric and Top5) with all other conditions the same. Fig 4 is a boxplot for the RMSD of the predicted models versus the experimental structures for the framework (FR) and whole Fv region including CDRs (FV), and Table 1 lists the *p* values which show whether the results are significantly different. These show that the Top5 method yields more accurate framework models which result in better models overall, and there is little difference between the Single and Chimeric approaches.

**Table 1:** Statistical comparison of framework modeling methods.

	FR *p* value	FV *p* value
**Top5/Single**	<0.001	<0.001
**Top5/Chimeric**	<0.001	0.005
**Single/Chimeric**	0.39	0.35

**Fig 5:**
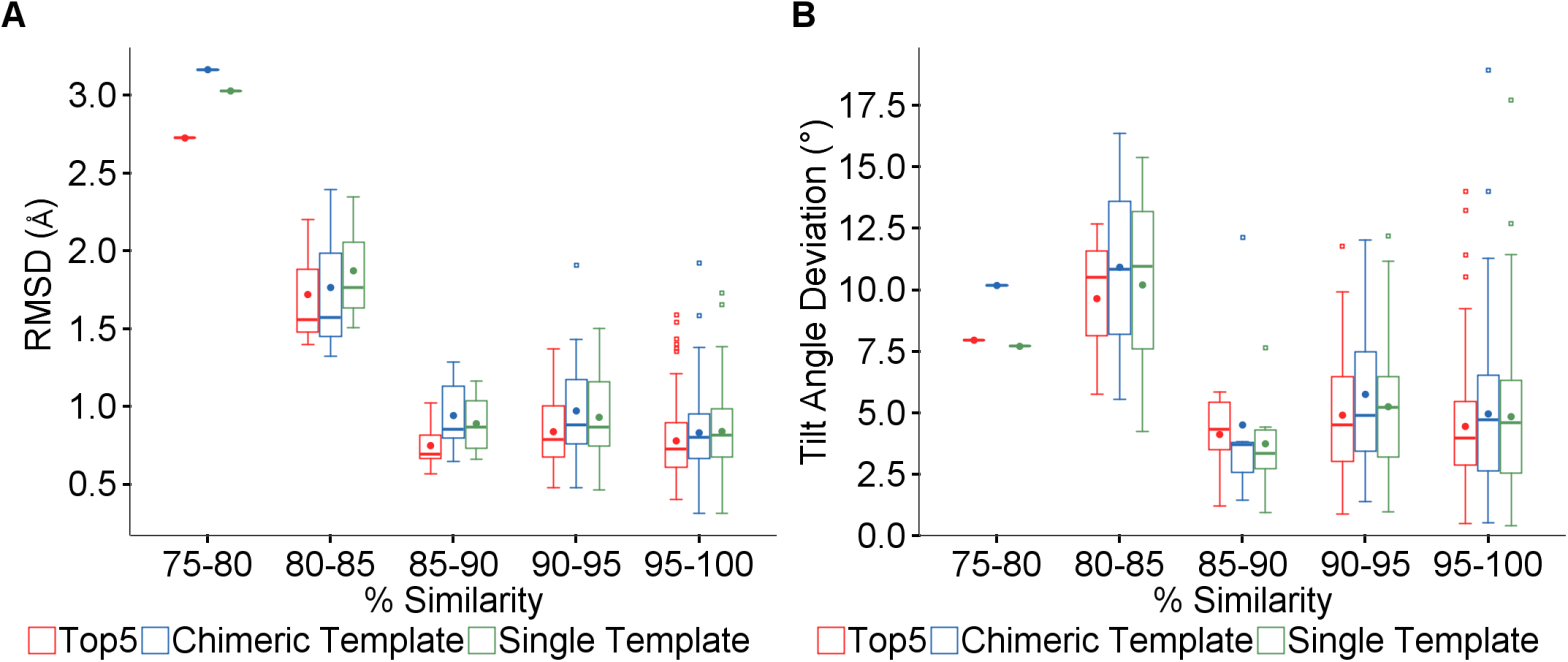
Effect of framework template similarity. (A) Boxplot of the RMSD binned by percentage similarity to the best template for the three framework modeling methods. (B) Boxplot of the tilt angle deviation.

As expected, the accuracy of the predicted model depends on the availability of sufficiently similar templates. Fig 5 shows the framework RMSD and tilt deviation for predictions using the Top5, Chimeric and Single methods. In each case, quite accurate results are generally obtained for all similarities above 85% but markedly worse below that, as shown in Table 2. All but four of the validation set do have at least one template in the database with >85% similarity.

**Table 2:** Statistics for similarities above and below 85% for the Top5 method.

	Number	Mean	Median	75^th^ percentile
RMSD (Å) <85% Similarity	4	2.0	1.9	2.3
RMSD(Å)>85% Similarity	144	0.8	0.75	0.9
Tilt Deviation(°) < 85% Similarity	4	9.2	9.2	11.0
Tilt Deviation(°) >85% Similarity	144	4.6	4.2	5.8

The similarity value used for the Top5 plot is that of the highest similarity template available, so it is interesting to note that while the similarity of the other four templates used may be lower, the models produced by this method tend to be more accurate than those using just the single best template even for very high similarities.

**Fig 6:**
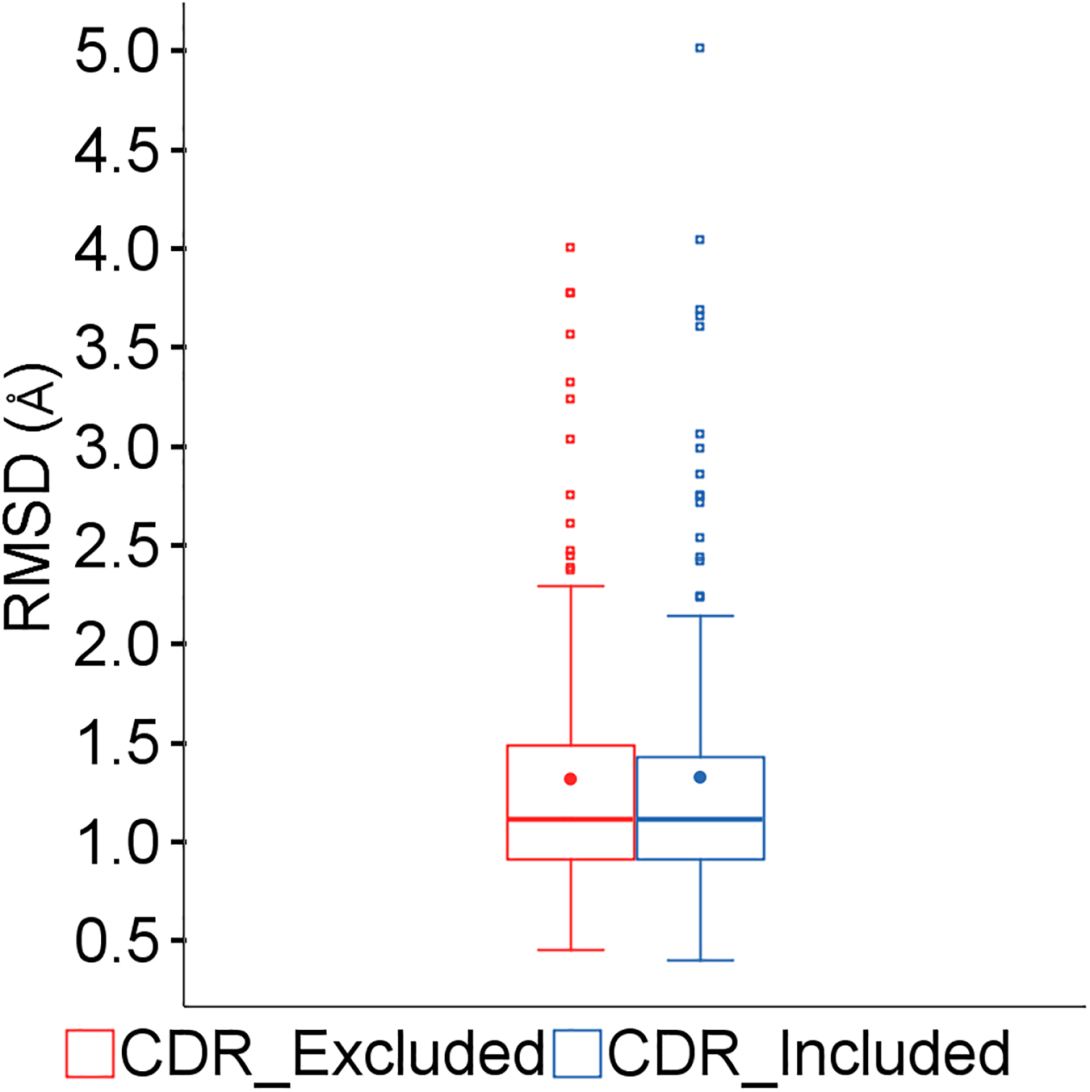
Effect of including CDR regions in similarity calculations.

The CDR loops are by default excluded from the similarity and identity calculations used to select the templates for framework modeling. Including them makes no significant difference to the overall accuracy of the models, as shown in Fig 6. However, as discussed below, there are some sequences for which including the CDRs is beneficial.

### CDR loop refinement

**Fig 7:**
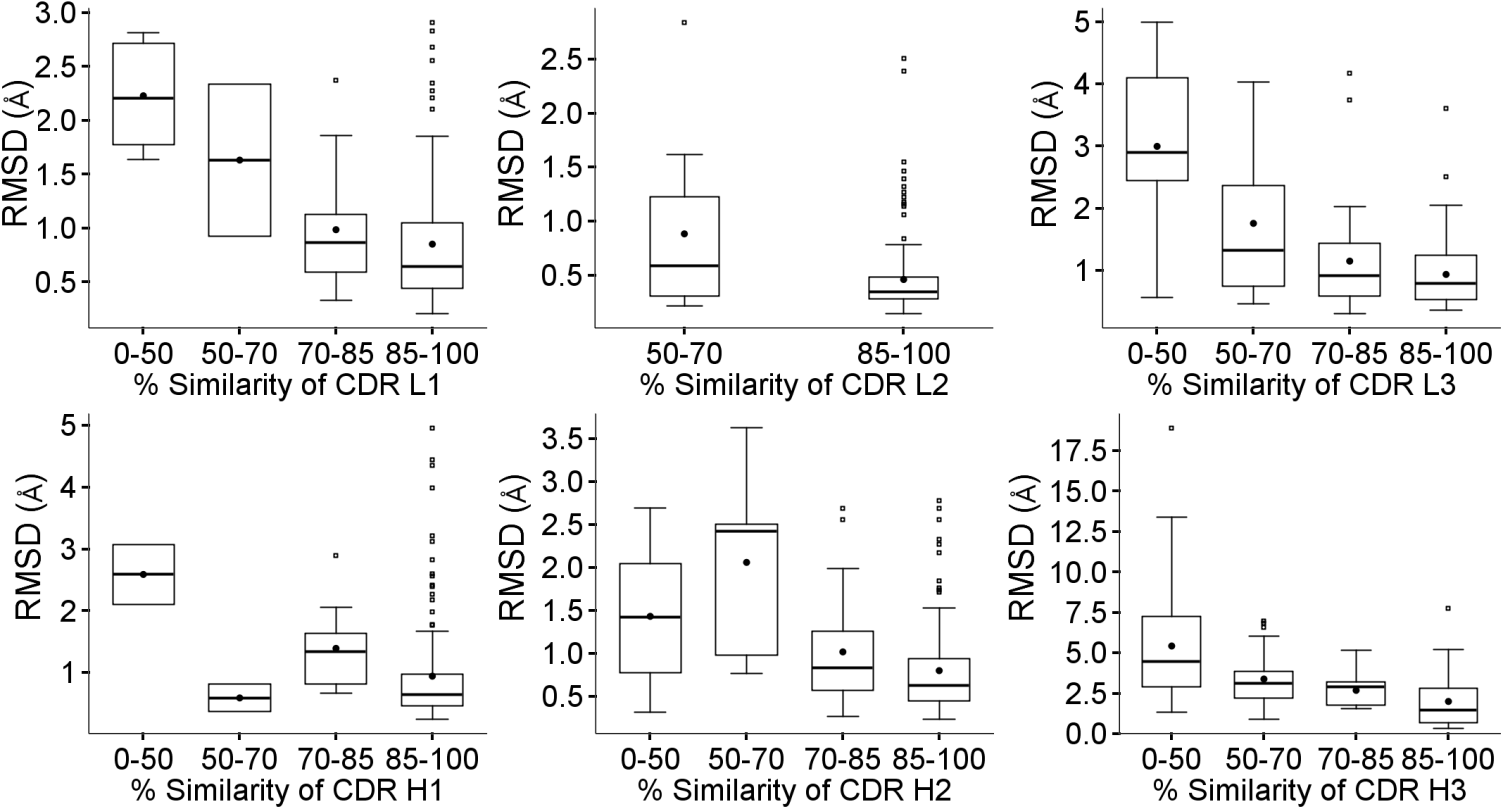
Effect of loop template similarity. Boxplots showing the effect of similarity of each CDR region on the RMSD for that loop.

The accuracy of the CDR loop modeling will depend on the quality of the initial framework model, as shown in Table 3, but also on the similarity of the loop templates. This trend is shown in Fig 7.

**Table 3:** Statistics for CDR RMSD similarities above and below 85% framework similarity.

	Mean RMSD(Å)		Median RMSD(Å)		75 Percentile RMSD (Å)	
CDR	Sim <85%	Sim >85%	Sim <85%	Sim >85%	Sim <85%	Sim >85%
L1	2.0	0.9	1.7	0.7	2.0	1.1
L2	0.9	0.5	0.9	0.3	1.2	0.5
L3	2.6	1.2	2.3	0.9	3.2	1.4
H1	1.1	1.0	0.9	0.7	1.7	1.1
H2	1.7	0.9	1.7	0.7	2.6	1.1
H3	8.6	3.7	9.8	3.0	11.1	4.8

It has been shown in previous studies that the accuracy of the loop models is related to its length [25]. This is particularly relevant in the case of H3.

**Fig 8:**
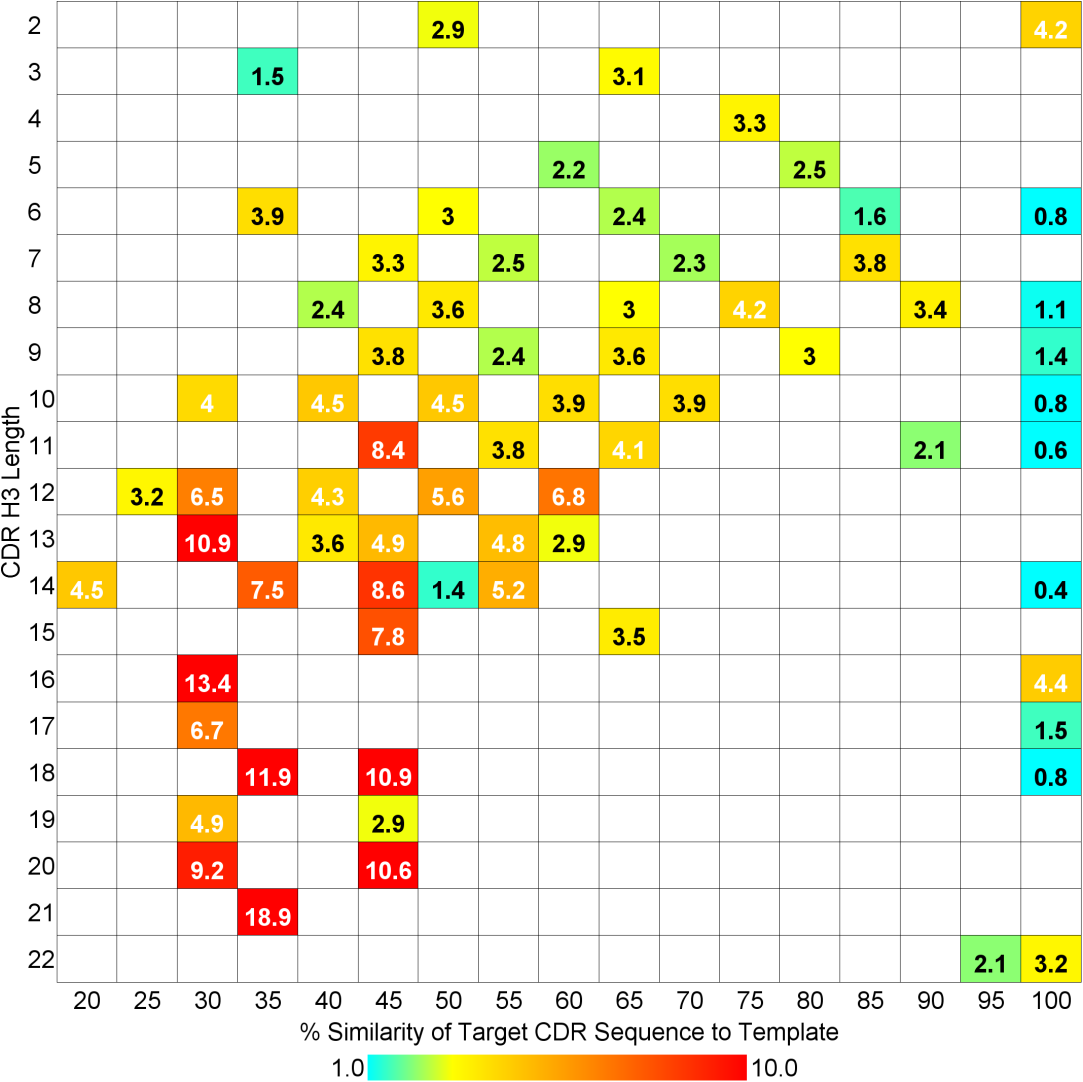
Heat map of average RMSD (Å) for CDR H3 length versus % similarity of best loop template.

However, even for quite long loops, reasonable models may be obtained if there is a highly similar template available. This is illustrated by Fig 8, which is a heat map of the average CDR RMSD for each H3 loop length/similarity combination present in the dataset. The Discovery Studio 4.1 database provided templates with a similarity above 85% for over a quarter of H3 loops with lengths greater than 13 (8 out of 29 cases); as the number of structures deposited in the PDB grows this should increase.

**Fig 9:**
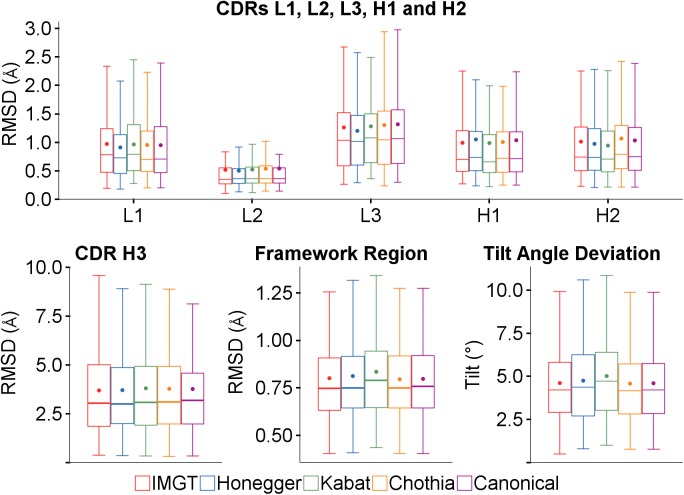
Effect of different loop definitions on RMSDs and tilt angle deviation.

The predictions were run for the sequence set using the IMGT, Honegger, Kabat, Chothia and Chothia with canonical filtering loop definitions, all other parameters being the same. In Fig 9, while there is some variation, there is no clear overall best choice. Comparing each of the other definitions against Chothia, the only statistically significant differences are that the framework RMSD and tilt angle deviations are slightly worse using the Kabat definition (*p* values 0.05 and 0.03).

To examine the effect of different template choices in more detail, we identified the cases where different sets of templates had been selected by the Chothia definition with and without canonical filtering. Those which had reasonable framework templates (similarity >85% and RMSD < 1.0 Å) and with loop RMSDs differing by more than 0.5 Å were analysed. The predicted structures are available in S1 Dataset and S2 Dataset.

#### CDR L1

Using the above criteria, in the comparison of canonical filtering against the unfiltered Chothia definition, there were two cases where canonical filtering was better than unfiltered (4LIQ_LH_FV and 4QWW_CD_FV) and one in which it was worse (4K7P_LH_FV).

**Fig 10:**
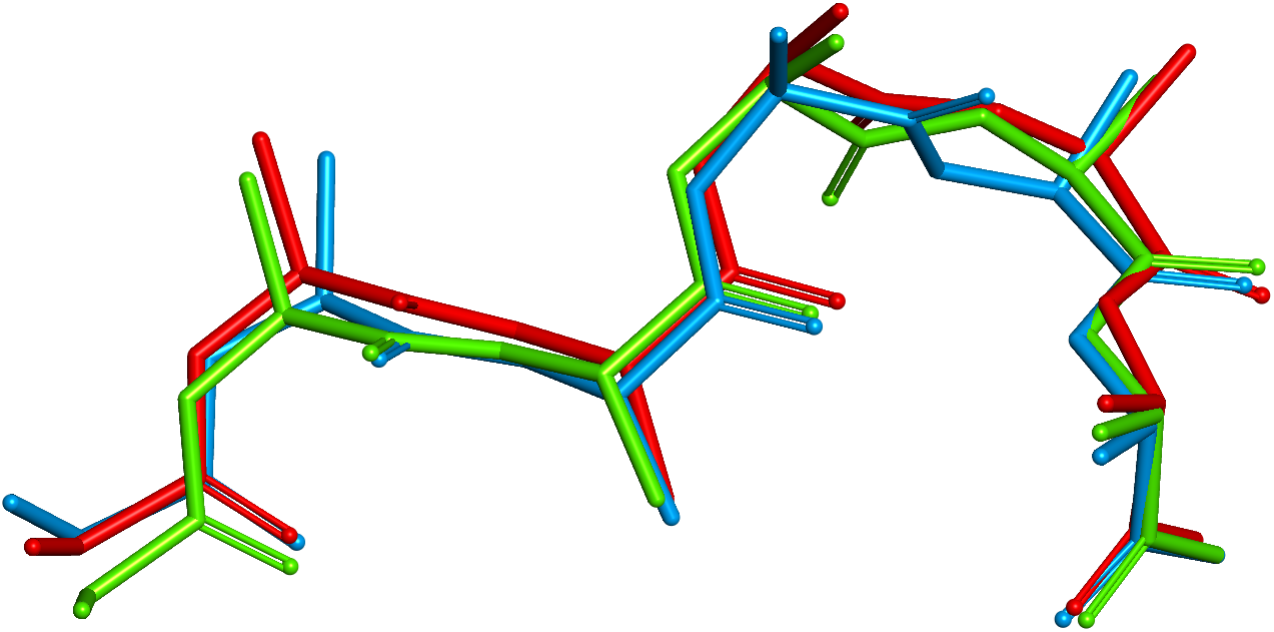
4LIQ CDR L1. X-ray structure red, prediction using canonical filtering green, prediction using unfiltered Chothia loop definition blue.

In 4LIQ, the choice of the correct kappa kL1:2A canonical for all three templates produces the correct conformation around the Asn30 residue with phi ~ 60° and psi ~ -120°, which is in a ‘marginal’ region of the Ramachandran plot. Without filtering, two of the selected templates are of canonical type kL1:2B which have phi/psi in ‘allowed’ regions but are not correct in this context. The choice was made on the basis of the scores including the stem regions, which were very slightly better; however, apart from being the wrong canonical type, the similarity and identity for the loop itself were lower. The effect, as shown in Fig 10, a plot of backbone and C-Beta atoms for the X-ray structure and the two predictions, is quite localised.

4QWW is rather more complicated. All the templates selected had the correct kL1:1 canonical type with or without the canonical filtering. In the case of filtering the templates selected were 4EBQ, 1AY1 and 1YQV. 1AY1 does not adopt the canonical conformation, with the Ser30 being flipped, however the predicted conformation was closer to the other two. The unfiltered prediction chose 4EBQ, 1AY1 and 3C09; the latter has better similarity than 1YQV but lower resolution. While its conformation is broadly similar to that of 4EBQ and 1YQV, favourable interactions with the L3 loop cause it to be displaced. The net result is that the predicted model tends towards the incorrect conformation of 1AY1.

The L1 loop of 4K7P is canonical kL1:2A. The templates of this type selected using filtering are 1MQK, 1F6L and 3V7A. However, the latter is flipped at Tyr30 relative to the other two; while the predicted loop lies closer to 1MQK and 1F6L at residues 29 and 31, it adopts the flipped conformation at residue 30. The templates chosen without canonical filtering are 1F6L, 2FR4, 1P7K. This set has a high ranking because they are also high scoring templates for the L2 and L3 loops. 2FR4 and 1P7K do not belong to any canonical type, but differ from type kL1:2A only in having a leucine rather than isoleucine at residue position 2 and adopt similar conformations to 1F6L.

The results for 4QWW and 4K7P suggest that it might be beneficial to check that the conformation of templates adheres to the canonical type.

#### CDR L2

**Fig 11:**
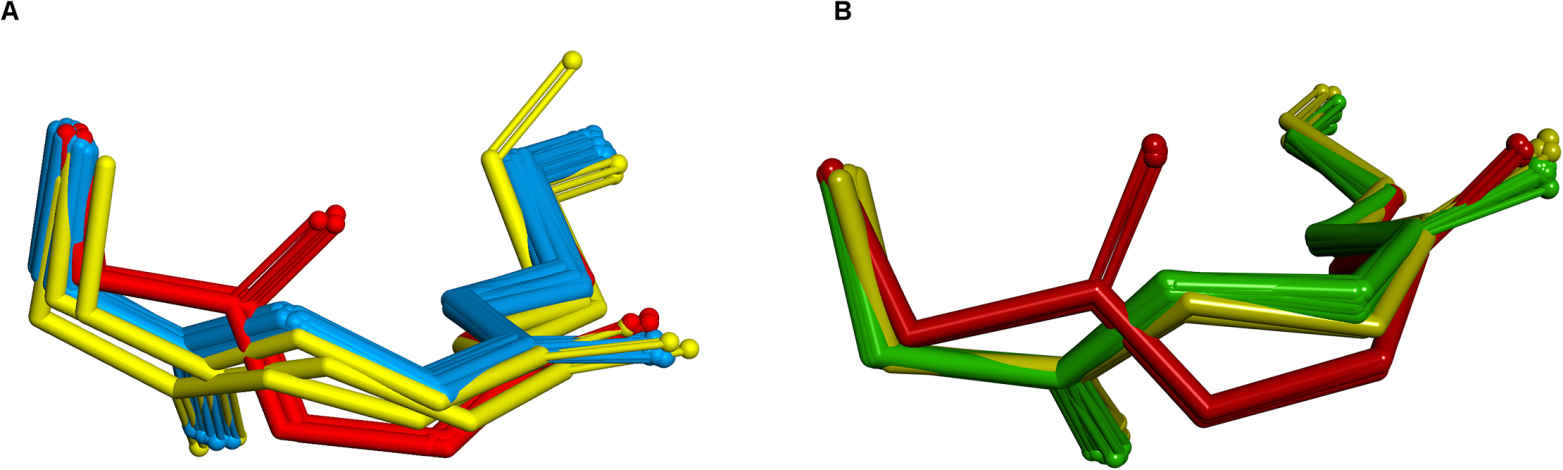
Predicted CDR L2 loop and templates for 4JO4 and 4C83. (A) 4J04; 3 anomalous conformations are shown in red; the other 47 in blue are close to the templates shown in yellow. (B) 4C83; 3 anomalous conformations are shown in red; the other 47 in green are close to the templates shown in yellow.

For the L2 loop, 4JO4_LH, which is one of the rabbit sequences, has canonical type kL2:1. With canonical filtering, the selected templates are 2CMR, 1LK3 and 1OP3. Without filtering, the latter is replaced by 1DFB. All templates are of the correct canonical type and have 100% identity. The conformations are all correct except for some deviation at residue 52 for 1DFB, which does not explain well why the predicted loop is flipped at the 50–51 peptide bond. Examination of a run in which 50 models were generated for each loop shows that this is an anomalous result, with only three of the models adopting the flipped conformation relative to the templates. This is shown in Fig 11A.

Conversely, in the case of 4C83_BA, accurate results are produced by the unfiltered method but there is a flip of the 50–51 peptide bond for the model obtained with filtering. The L2 loop of 4C83 is of type kL2:1 and the canonically-filtered templates are 2W9D, 4F33 and 4I9W. Without filtering, the latter is replaced by 3NCY which is also of the correct canonical type. As before, all the templates are in the correct canonical conformation and are 100% identical. Examination of a run with canonical filtering generating 50 models again shows that most do adopt the same conformation as the template but three are flipped. This is shown in Fig 11B. So, it seems that the differences in these cases are not really due to the method of choosing the templates but are an artefact of model building.

**Fig 12:**
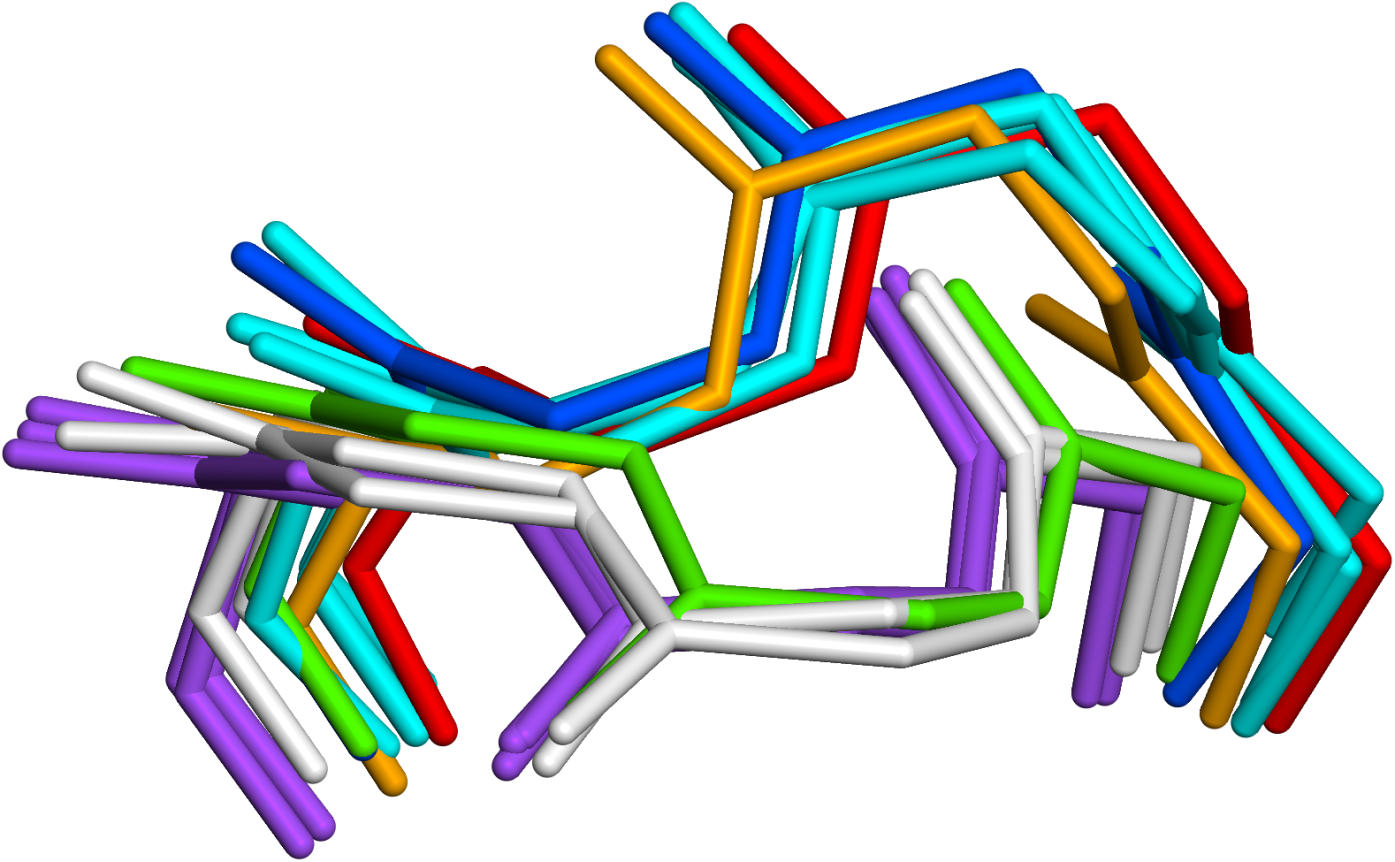
4JG1 L2 loop. X-ray structure red, prediction with canonical filtering green, prediction with no filtering blue. Templates with no filtering cyan; 4D9Q orange; 2ADF and 1FJ1 white. Some typical kL2:1 loops are shown in purple for comparison

4JG1_LH is a case in which canonical filtering yielded a poor set of templates. The canonical type is kL2:1, which requires isoleucine or valine at residue 48 and glycine at 64 The templates matching this type which were selected were 4D9Q, with a similarity and identity of 67%, while the other two, 2ADF and 1FJ1, had no similarity at all for the three loop residues. This pair adopted the conformation typical for the canonical whereas 4D9Q did not. Without filtering, the three templates selected (3BQU, 1I8K and 3I9G) had similarities of 100% with two being 100% identical. This set of templates had similar conformation to 4D9Q and correctly modelled the loop. However, they differed from the canonical definition by having a serine instead of glycine at residue 64. These are shown in Fig 12 together with some examples of typical kL2:1 canonical loops for comparison.

#### CDR L3

There were no cases found where the RMSD for the L3 loop differed by more than 0.5Å between the filtered and unfiltered templates.

#### CDR H1

4M5Y_LH benefits from canonical filtering, by selecting templates 3CX5, 4HC1 and 2XA8 which are all of the correct canonical type, H1:2. The templates have reasonable similarity and identity to the target, and adopt the same conformation. Without filtering, the first two of these are selected but the highest ranked template is 2XZC, which has a very high score because of high identity of the loop and stem regions. But it does not conform to any canonical type and adopts a different conformation which dominates the prediction.

In the case of 4O02_LH, all the selected templates were of canonical type H1:1 and had 100% similarity, 71.4% identity and a score of 89. They were 1WT4 in both cases, plus 3CMO and 1EHL with filtering. The unfiltered selection, which includes in its ranking criteria the scores of the other two loop regions, chose 1A6V_I and 1A6V_J. All the templates had the correct canonical conformation except for 1A6V_J, which differed at residues 29–30; the predicted model was similar to this. 1AV6 is a structure which contains three non-crystallographically related copies demonstrating the variability in conformation which can arise due to packing. Comparing the loops, the main-chain RMSDs are between 1–1.7Å for H1, 1.3–1.8Å for H2 and 1.1–1.5Å for H3.

4LEO_BA has a >0.5Å worse RMSD for the H1 loop for the prediction using canonical filtering versus unfiltered Chothia, but neither is very accurate (1.7Å and 1.2Å). The templates (3EO0_B and 1MJ8_H for both, plus 1MH5_B with filtering and 2UYL_B without) all had similarity/identity of 71%; this is relatively low compared to most of the other cases.

#### CDR H2

**Fig 13:**
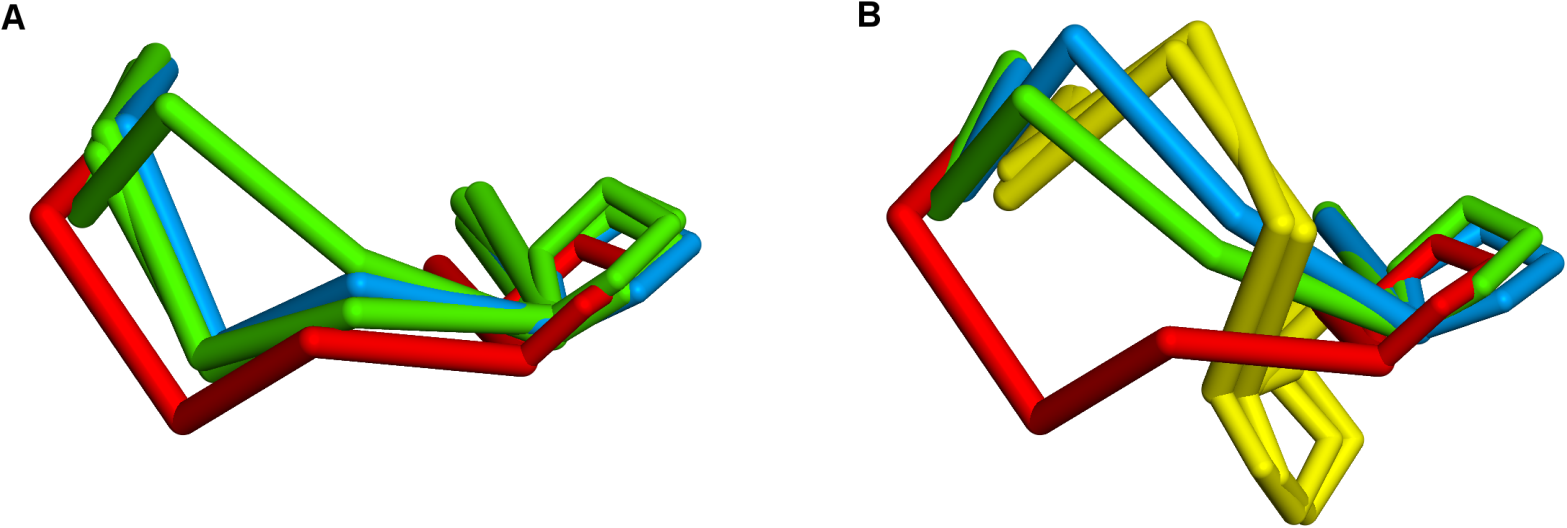
4NKI_LH H2 loop. X-ray structure red, predicted structure blue, H2:3 templates green, H2:2 templates yellow. (A) with canonical filtering. (B) without canonical filtering.

4NKI_LH is a case where using canonical filtering yielded a much better result for H2 versus the unfiltered Chothia definition (RMSD for the loop residues 0.4Å vs 2.4Å). The target sequence has canonical type H2:3. With filtering, the selected templates were 3HI6_H, 3HI5_H and 3KYM_B (Fig 13A), whereas without filtering the templates chosen were 3HI6_H and 3K2U_H, 2WUC_H, the last two of which are canonical H2:2 (Fig 13B). The reason for this choice was that they have slightly better scores for the loop region including the stems; however they adopt a significantly different conformation especially at Pro52A.

In 4G80_BA, the canonical type is H2:2, and the templates chosen using filtering are 1NJ9_B, 3EFD_H and 3IVK_A. The latter adopts an anomalous conformation at Pro52A but the prediction is closer to the other two; this produces a good fit, with a loop RMSD of 0.66Å. Without filtering, the templates are again 1NJ9_B, 3IVK_A and the H2:3 canonical 1SEQ_H. In addition to being the wrong canonical type, 1SEQ_H also has similarity/identity of only 25% but has a better score including stem regions than 3EFD. It has a drastically different conformation and the resulting prediction, having only one template with a typical canonical conformation, is inaccurate with a loop RMSD of 1.56Å.

### Filtering by organism

The protocol allows the choice of templates for both the framework and CDR loops to be restricted to those from a specified organism. Running a prediction on just the sequences classified as human, with canonical filtering and specifying the organism as ‘human’, 47 sequences had at least one loop with no templates found, of which 23 had no loops matched and so no model created. Predictions on the sequences classified as mouse with filtering by organism ‘mouse’ resulted in 37 cases where at least one loop could not be modelled. Combining the results for which all loops were predicted using organism filtering and comparing against the prediction with no organism filtering showed little overall difference, with some loop types being rather worse with the filtering. So in general it does not appear to be beneficial to use this option. It should be noted that the taxonomic classification will not be correct in the case of engineered antibodies, as one or more loops may not derive from the organism of the rest of the structure.

### Effect of number of cycles of refinement

**Fig 14:**
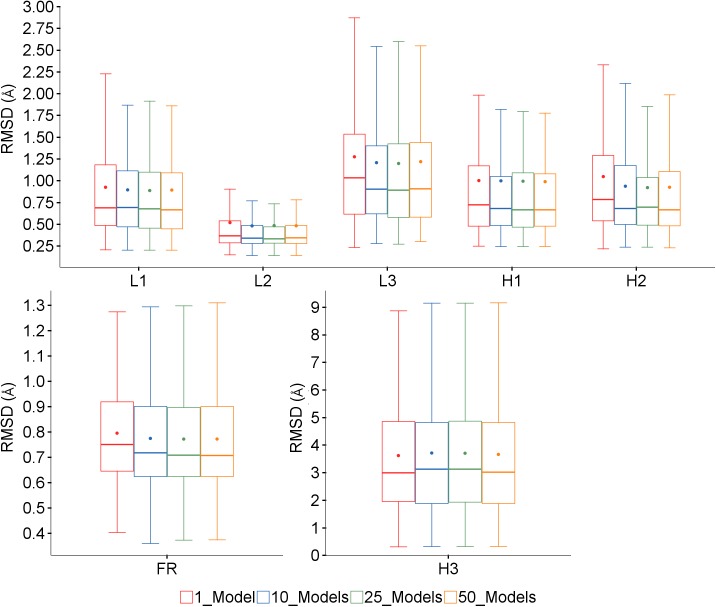
Effect of number of cycles of refinement. Box plots showing variation in framework and loop RMSDs for different numbers of framework models and CDR refinement cycles.

The predictions were run to generate N framework models using the Top5 method, and then for the best of these, N loop models using the Chothia definition, for N = 1, 10, 25 and 50. Fig 14 shows that very little difference can be seen between the overall results for the framework or loop regions. There is a slight improvement for the framework and loop regions except for H3 on increasing from 1 model to 10, but little change thereafter. The differences are only statistically significant for the framework region and loops L2 and H2, and probably not large enough to be meaningful in practice.

### Examination of outliers

In order to understand factors affecting the accuracy of the predicted structures, outliers were examined to see what might be giving rise to unusually large discrepancies from the X-ray structures in a small subset of cases. As is clear from Fig 5, a major consideration is whether high similarity templates are available in the database. To account for this, the analysis was performed considering only those for which there was an overall Fv template with similarity above 85%. In addition, cases without loop templates of the correct length are also likely to be unreliable so these were excluded from this part of the analysis. The predictions generated using the Top5 framework template method and Chothia loop numbering were used for the analysis; these structures are available in S1 Dataset.

#### Framework region

**Fig 15:**
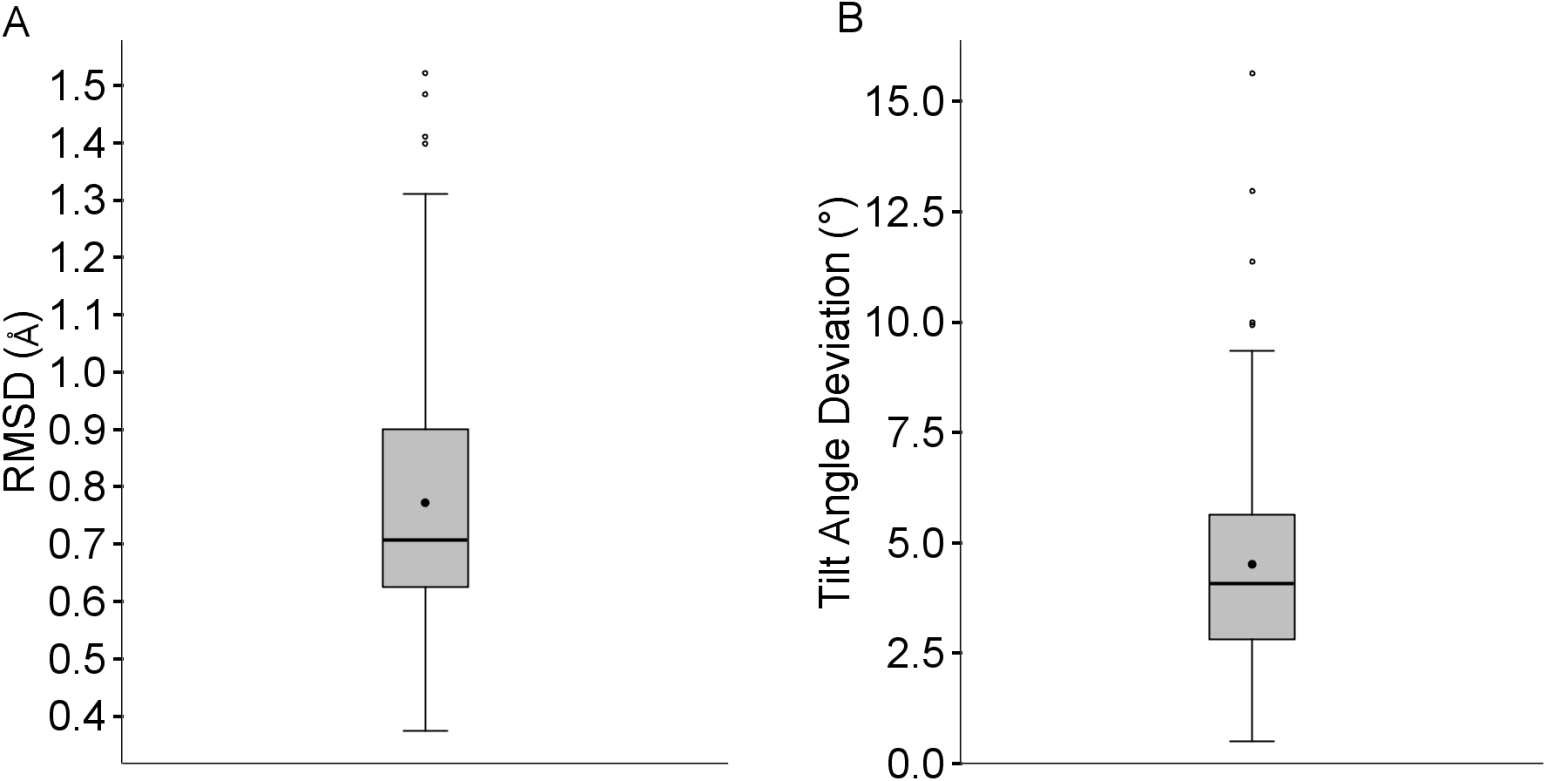
RMSD and tilt angle deviation for predictions with good templates available.

Fig 15A shows that the framework RMSD for 75% of the predicted structures is within 0.9Å of the experimental structure, with a median value of 0.7Å and all below 1.5Å. The four outliers with RMSDs above 1.3Å are listed in Table 4: 3ZL4_LH (1.5Å), 4QHM (1.5Å), 4LVH_CB (1.4Å), 4QHM (1.5Å) and 4MWF_LH (1.4Å). Fig 15B shows that the tilt angle deviations are generally quite low, with 75% falling below 6°. There are five outliers with angles above 9° shown in Table 5: 3ZL4_LH (15.6°), 4CNI_BA (13°), 4NIK_BB (11.4°), 4MWF_LH (10.0°) and 4FZE_LH (9.9°). Unsurprisingly, some structures are outliers for both RMSD and tilt deviation.

**Table 4:** Framework RMSD outliers.

Name	RMSD(Å)	Tilt(°)	Identity(%)	Similarity(%)	Templates
3ZL4_LH	1.5	15.6	77–97	83–98	2XZC_LH,3MLY_LH,3MLX_LH,2J6E_LH,2YK1_LH
4QHM_BA	1.5	8.1	64–89	80–96	4JAM_LH,4JAM_BA,4FQQ_CD,4FQQ_AB,3GO1_LH
4LVH_CB	1.4	4.2	69–96	82–97	4F15_CB,2GCY_AB,2OR9_LH,2ORB_LH,2ORB_MI
4MWF_LH	1.4	10.0	67–82	81–91	4DN3_LH,4DN4_LH,1RHH_AB,1RHH_CD,1G9M_LH

**Table 5:** Tilt angle deviation outliers.

Name	RMSD(Å)	Tilt(°)	Identity(%)	Similarity(%)	Templates
3ZL4_LH	1.5	15.6	77–97	83–98	2XZC_LH,3MLY_LH,3MLX_LH,2J6E_LH,2YK1_LH
4CNI_BA	1.2	13.0	73–87	82–95	3SKJ_LH,2KH2_BB,2FJH_AB,2FJF_AB,3BDY_LH
4NIK_BB	1.2	11.4	69–84	83–92	3TNN_BA,3H42_LH,4F58_LH,4F57_LH,1NL0_LH
4MWF_LH	1.4	10.0	67–82	81–91	4DN3_LH,4DN4_LH,1RHH_AB,1RHH_CD,1G9M_LH
4FZE_LH	1.1	9.9	67–82	80–90	3MA9_LH,3NPS_CB,4JZO_BA,4JZO_FC,3MAC_LH

The best ranked template for 3ZL4_LH is 2XZC_LH. The sequences of the Fv regions have a very high identity, differing only in the last four residues of the L chain and one near the end of the H chain. Superimposing the X-ray structure onto this template and calculating the framework RMSD gives a value of 1.7Å, similar to the discrepancy in the predicted model. However, examination of the full sequence shows that the light chain of 3ZL4 has lambda variable and constant domains, whereas in 2XZC there is a kappa constant domain[26]. The structure was engineered in order to investigate the effect of switching between kappa and lambda constant domains on the structure and functionality of the antibody; this was found to cause a 12° change in the elbow angle. If the structure of the full Fab domain is predicted, all of the templates used for the framework model have a lambda constant domain and the RMSD for the framework of the Fv region of this structure falls to within 1.1Å of the X-ray structure.

Examining 4LVH_CB, the discrepancies in the framework lie mainly at the N-termini of the L and H chains, which appear to be misaligned by one residue. The misalignment in the L chain occurs around Pro8, which is in the trans form in the X-ray structure but adopts the cis conformation in the predicted model. The most similar template has a trans Pro8 but the other four are cis. In the case of the H chain, the discrepancy arises in the region before Gly8, Gly9 and Gly10. This highly flexible region allows for variation in the templates which is reflected in the model structure. An examination of the experimental structure for violations shows that there are 45 non-planar peptide bonds whereas there are none in the predicted structure; comparison of Ramachandran plots similarly shows fewer violations in the predicted structure. It is unsurprising that a modeled structure does not replicate these violations.

In 4QHM_BA, the most obvious structural difference is in the turn between Gln39 and Leu45 of the heavy chain. Examining the relationship to the templates, it is evident that while the overall similarity to the target is within 10% of that of the best template, the discrepancy is greater if the CDR regions are also considered. This is shown in Table 6. In this case the inclusion of the lower-similarity templates appears to be leading to sub-optimal modeling of some regions; the single template method yields a lower framework RMSD (1.0Å). However, the overall RMSDs for the models produced by the two methods are very similar (1.49Å and 1.42Å). Better results (framework RMSD 1.0Å, overall RMSD 1.1Å) are obtained in this case by not excluding the CDR regions from the similarity and identity calculation; the top 5 overall templates are still the same but only the first two are used as the similarities of the others are over 10% poorer.

**Table 6:** Similarities for the 4QHM_BA templates.

Template	Similarity(no CDR)	Identity(No CDR)	Similarity(All)	Identity(All)
4JAM_LH	96.3	89.4	93.4	87.3
4JAM_BA	95.2	88.4	90.8	84.7
4FQQ_CD	88.4	79.4	81.6	68.6
4FQQ_AB	88.4	79.4	80.9	68.1
3GO1_LH	87.8	73.0	79.9	63.8

4MWF_LH is an outlier both for RMSD and tilt angle deviation. The H3 loop is 16 residues long and includes a disulfide bridge. It adopts a significantly different conformation from the H3 loops used to build the framework model, and even more different from any of the templates used for CDR modeling, which only have similarities of 25–31%. It is likely that this large discrepancy in the final loop conformation causes the inaccurate orientation of the domains.

4CNI_BA and 4FZE_LH may be outliers for tilt angle because they adopt VL-VH orientations towards the extremes of the distributions found using the ABangle webserver [27] for at least one of its measures. In the case of 4FZE, this is particularly marked for the HL angle, whereas for the template 4JZO (which is used twice as it exists in two slightly different forms in the crystal structure) this angle lies towards the other extreme. Using the single template method for framework prediction with the template 3MA9 yields a much more accurate structure with tilt deviation 3.7°, framework RMSD 0.6Å and overall RMSD 1.5Å.

#### Loop regions

**Fig 16:**
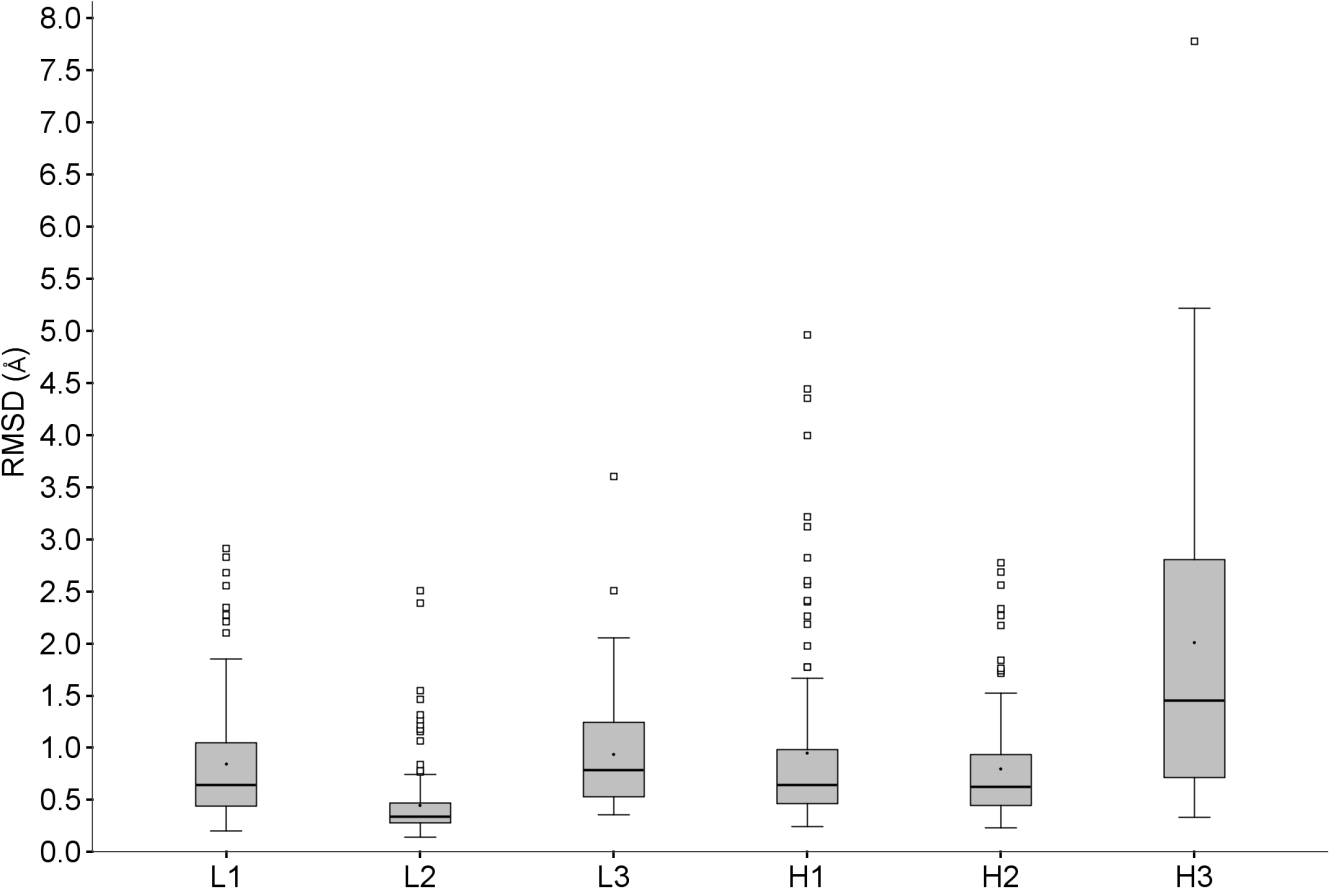
RMSDs for CDRs for predictions with good templates available.

The loop regions were examined in a similar way to identify factors other than template similarity which could adversely affect accuracy. This part of the analysis was therefore further restricted to the cases for which the loop being investigated had at least one template with similarity above 85%. The RMSD ranges for these are shown in Table 7 and Fig 16.

**Table 7:** RMSD statistics for the CDR loops.

	L1	L2	L3	H1	H2	H3
Median RMSD(Å)	0.6	0.3	0.8	0.6	0.6	1.5
75 Percentile RMSD(Å)	1.1	0.5	1.2	1.0	0.9	2.8
Number of Outliers	8	13	2	16	10	1

#### CDR L1

In the L1 outliers shown in Table 8, the deviation in 4HIE_AB arises from the templates having a significantly different conformation for residues 30 and 31. These residues are SS in all the templates but TN in the target sequence. The Ser30 residues of the templates have backbone angles which are not in the allowed region of a Ramachandran map. This conformation may be stabilised by non-bonded interactions e.g. a hydrogen bond between Ser31 and Asp50 and Tyr32 whereas the Asn31 makes completely different interactions with Arg91, Asp92, and Phe100A.

**Table 8:** Outliers for CDR L1.

	RMSD(Å)	Length	Residues	Identity(%)	Similarity(%)	Templates
4NIK_BB	2.9	11	GTSSDVGGYNY	100	100	4F57_L,2MCG_1,1MCS_B
4IOF_FE	2.8	7	SQSVSSA	100	100	2R8S_L,3PNW_V,3PNW_A
4NKI_LH	2.7	11	GTSSDVGGYNY	100	100	4F57_L,2MCG_1,1MCS_B
4HIE_AB	2.6	7	SQSVTNY	71	86	3GIZ_L,3EYQ_C,3F12_A
4O4Y_LH	2.4	9	SQSVYNKNY	22–78	56–89	4J1U_A,3UTZ_D,1QFW_L
4FZ8_LH	2.3	12	SQSLLHSNGYNY	67–75	92–100	3NZ8_B,3QEH_H,3QEH_B
4K94_LH	2.2	7	SQSVSSA	100	100	2R8S_L,3PNW_V,3PNW_A
4IDJ_LH	2.1	8	SQTISNNF	38–50	75–88	2V7N_A,4HWE_L,4FQL_L

4NIK_BB is another example of a fairly long loop (11 residues) containing 3 glycines; although the sequences are identical, there is a high degree of variability in the loop conformation of the template and target crystal structures.

#### CDR L2

The outlying L2 loops are listed in Table 9; note that while there seem to be a large number of them, the median and 75 percentiles are lower than for other loops. The L2 loops are typically very short using the Chothia and IMGT definitions, so the cases with outlying RMSDs are likely to arise from misalignments in the stem region. This is the case for 4O02_LH in which the loop is defined as residues 50–52 and the RMSD (which is calculated for 50–53) is 2.5Å for Chothia and similar for IMGT. The framework templates deviate from each other at residue 53, which results in the prediction similarly deviating from the experimental structure. It is worth noting that the values for Kabat which extends the loop to 7 residues and Honegger which has 12, the RMSDs are 0.8Å and 1.0Å respectively; the framework templates align better and so the loop can also be placed more correctly.

**Table 9:** Outliers for CDR L2.

Name	RMSD(Å)	Length	Residues	Identity(%)	Similarity(%)	Templates
4O02_LH	2.5	3	YTS	100	100	4G6M_L,1FNS_L,1YNT_A
4K7P_XY	2.4	3	APS	67–100	67–100	3BKY_L,2OSL_B,1AD0_A
3WHE_09	1.6	3	GNS	67–100	67–100	3H42_L,4D9L_L,2JB5_L
4U1G_CB	1.5	3	YAS	67–100	100	1NSN_L,1I9R_L,3U9P_L
4N9G_NM	1.3	3	YTS	100	100	1FGV_L,4JR9_L,3ESU_F
4IOF_FE	1.3	3	STS	67–67	100	2R8S_L,3PNW_V,3PNW_A
4KVC_LH	1.2	3	GAS	100	100	1UYW_L,1IGC_L,1IGY_A
4QHN_BA	1.2	3	ENY	0–100	100	4JAM_L,4JAM_B,3MLR_L
4IDJ_LH	1.2	3	GAS	100	100	4FQL_L,1RHH_A,1IQD_A
4PY7_BA	1.1	3	AAS	100	100	3NCJ_L,3BN9_C,2R56_L
4M1G_LH	0.8	3	RTS	67–100	67–100	3LS5_L,1MIE_L,1AE6_L
4NKO_AB	0.8	3	KVS	100	100	2GK0_A,1I9J_L,3NN8_B
4LVH_CB	0.8	3	TAS	67–100	100	4F15_C,1H0D_A,2HRP_L

In the case of 4K7P_XY, the discrepancy occurs because the target sequence has a proline at residue 51 of the loop region which adopts the cis conformation in the crystal structure. However, none of the framework templates has a proline in this position and so are all in the trans configuration; of the CDR templates, only one has proline and it too is trans. Even if the CDR regions are included in the framework template similarity, only one has a Pro51 and it is not in the cis conformation.

4U1G_CB is a case where the predicted and experimental loops appear reasonably similar but both have main chain torsion angles which are not in the allowed regions of a Ramachandran plot–but for different residues. This leads to different placement of the carbonyl group and sidechains in the loop. The distortions in the PDB structure of 4U1G_CB are quite extreme. None of the CDR templates adopt its unusual main-chain conformation at Tyr50 (although the tyrosine ring is in a similar position) so it seemed possible that this loop was incorrectly placed in the crystal structure. Therefore, the re-refined structure from pdb_redo (http://www.cmbi.ru.nl/pdb_redo/) was examined; this was closer to the predicted structure, with an RMSD for the loop of 1.0Å.

#### CDR L3

**Fig 17:**
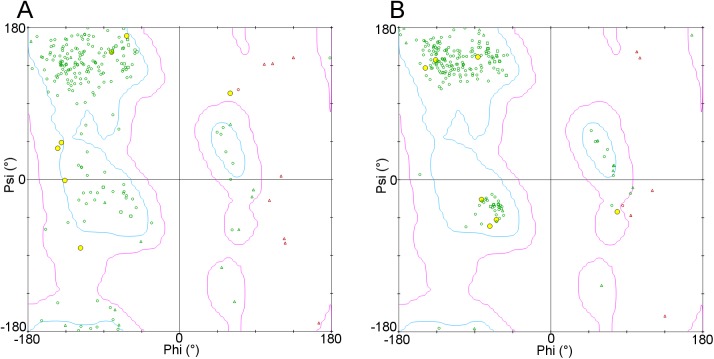
(A) Ramachandran map of the 4QHK_NM X-Ray structure. (B) Ramachandran map of the predicted structure for 4QHK_NM. For both plots, the cyan line contains the favourable regions and the magenta line the marginally allowed regions. Proline residues are marked by squares, glycine by triangles and other residues by circles. Residues falling in the disallowed regions are coloured red. The residues of the L3 loops are highlighted in yellow.

The outliers for CDR L3 are shown in Table 10. The L3 loop of 4QHK_NM has an RMSD of 3.6Å. The difference between the prediction and the crystal structure arises from the presence in the latter of two non-proline cis-peptides in the loop, Asp92-Ser93 and Phe94-Ser95. In addition, most of the main-chain angles are in unfavourable regions of a Ramachandran map, shown in Fig 17A. There are no cis-peptides in any of the framework or CDR templates used to model this structure and fewer main-chain violations in the prediction, as shown in Fig 17B. It seems to be a case in which the prediction is plausible but the experimental structure is in an unexpected conformation.

**Table 10:** Outliers for CDR L3.

Name	RMSD(Å)	Length	Residues	Identity(%)	Similarity(%)	Templates
4QHK_NM	3.6	7	WDSFSTF	57–100	71–100	4JAM_B,4JAM_L,2G75_B
4LVH_CB	2.5	6	TKEVPY	67–83	100	2ORB_L,2OR9_L,2HRP_L

#### CDR H1

Table 11 lists the outliers for the CDR H1 loop. The predicted H1 loop of 4NNP_LH deviates significantly from the observed structure. Examining against the framework templates, the difference in the region around Ser31 may be due to interactions between the H1 and H3 loops–the latter is 17 residues long in the target structure whereas that of the templates are only 7 or 10 and adopt a quite different conformation. In all cases there are hydrogen bonds between the two loops. There is no obvious reason for the marked difference at Gly26; the flexibility of this residue presumably allows the remainder of the loop to adopt a different conformation as the result of crystal packing.

**Table 11:** Outliers for CDR H1.

Name	RMSD(Å)	Length	Residues	Identity(%)	Similarity(%)	Templates
4NNP_LH	5.0	7	GFNFSSS	71–100	86–100	2QR0_B,3PGF_H,2R8S_H
4OGY_LH	4.5	7	GFTFSHY	86–100	100	3SKJ_H,3KR3_H,2ORB_H
4MWF_LH	4.5	7	GGTFDNY	86	86	1RZG_C,1RZG_A,2QAD_D
4KXZ_LH	4.0	7	GYTFSSN	86–100	100	3EO0_B,1JFQ_H,6FAB_H
4QHK_NM	3.2	7	GGSISSY	86–100	100	4FQQ_D,4FQQ_B,1U6A_H
3W9E_BA	3.1	7	GGTLRTY	57–86	86	1LO4_H,4DN3_H,4DN4_H
4M62_LH	2.8	7	GGTFSSY	100	100	3NPS_B,3QOT_H,4HJ0_C
4JO4_LH	2.6	8	GFSFTNNY	50–75	75–100	1ORS_B,2AJU_H,3CX5_J
4KQ3_LH	2.6	7	GGTFSSY	100	100	3QOT_H,1RZI_B,4HJ0_C
4NWT_LH	2.4	7	GGTFSNY	100	100	1RZG_A,1RZG_C,2QAD_D
4N90_ED	2.4	9	GGSISSGDY	67–100	78–100	3B2U_C,2VXQ_H,3TNM_H
4KI5_DC	2.3	7	DYTFTDY	86	86	2W60_A,3MO1_B,3MNZ_B
4FZE_LH	2.2	7	RGTLSSY	71	86	3NPS_B,3QOT_H,2G75_A
4QHM_BA	2.0	7	GGSMGGY	57–86	71–86	4JAM_H,4FQQ_D,4FQQ_B
4R2G_MN	1.8	7	GGSISNY	71–86	86	4FQQ_D,4FQQ_B,3FN0_H
4OCX_LH	1.8	8	GFSITSPY	75	88	3G5Y_B,1KCU_H,1KCR_H

#### CDR H2

Table 12 lists the outliers for the CDR H2 loop. 4OGY_LH is an outlier for both the H1 and H2 regions. The framework templates show good agreement to the X-ray structure for both the H1 and H2 stem regions. For the H1 loop, the templates all adopt a similar conformation to each other but differ from the target X-ray structure; again, there is a Gly26 residue. The H2 loops all have Gly54 and Gly55. In fact, of the outlying H1 and H2 loops, all but one contains one or more glycine residues.

**Table 12:** Outliers for CDR H2.

Name	RMSD(Å)	Length	Residues	Identity(%)	Similarity(%)	Templates
4NKI_LH	2.8	4	PSGG	75–100	100	3K2U_H,2WUC_H,3HI6_H
4OGY_LH	2.7	4	SSGG	50–100	75–100	3GJF_H,4GLR_H,3K2U_H
4MWF_LH	2.5	4	PLFG	75	100	3GBN_H,4EVN_A,1RHH_B
4BZ2_LH	2.3	4	PYNG	75–100	75–100	1UM5_H,2PCP_B,1F3D_H
4LF3_AB	2.3	4	YDGS	75–100	75–100	4ERS_H,4G5Z_H,3EYQ_D
4I18_BA	2.2	4	PYYG	75–100	75–100	3DVN_B,3DVG_B,3EFD_H
4LLW_BA	1.8	4	PNSG	75–100	75–100	1L7I_H,3CVI_H,1KTR_H
4KXZ_LH	1.8	4	PIVD	25–100	75–100	3EO0_B,3QEG_H,3LH2_I
4LVH_CB	1.7	4	GGSE	50–100	75–100	4F15_B,2Q76_B,1P2C_B
4K94_LH	1.7	4	PYSG	100	100	2FJH_B,3IVK_A,1ZA3_B

4NKI_LH is the case where using canonical filtering will yield a much better result for H2 as discussed in detail in the Comparison of Loop Definitions section.

#### CDR H3

The H3 loops show much more variability than the others, as is expected. Examining the one outlying case 4LLV_LH, which is shown in Table 13, it is noted that the crystal structure contains other non-crystallographically related heavy chains. Comparing their H3 loops, the RMSDs are 4.3Å and 1.6Å, showing that there is a significant degree of conformational flexibility possible for this loop, which contains four glycine residues and is sixteen residues long. The loop templates have very high identity with the target sequence (2FX7 is identical, the other two only differ by one residue) and all adopt a very similar conformation to the predicted model. It seems likely that the conformation is driven by the local packing environment in the crystal, which is markedly different between 4LLV and 2FX7, and could well be quite different in a non-crystalline environment.

**Table 13:** Outliers for CDR H3.

Name	RMSD(Å)	Length	Residues	Identity(%)	Similarity(%)	Templates
4LLV_LH	7.8	16	GTTGWGWLGKPIGAFA	94–100	94–100	2FX7_H,3LH2_K,3LH2_J

## Conclusions

In general, the automated antibody structure prediction can produce models with quite accurate framework regions (RMSD typically below 0.9Å and tilt angle deviation below 6°) and reasonable CDR loops for all but H3, as shown in Table 7. The Top5 framework method produces small but significant improvements in model accuracy, but the predictions are not particularly sensitive to the other parameters. Of the various combinations of parameters examined, the best overall results were obtained using the Chothia scheme with canonical filtering, with 5 CDR templates and 10 CDR models generated, and using the default values for other settings. While the differences between the various sets of results obtained using the Top5 framework method and varying other parameters are statistically insignificant, the use of more templates and the generation of more models can be helpful in some cases. The predictions using these parameters are available in S3 Dataset.

The accuracy of the models depends heavily on the availability of appropriate templates for the framework and loop regions. The number of such templates continues to grow steadily; whereas the database used for this validation contained a total of 4950 non-redundant domain sequences, the latest Discovery Studio 2016 release has 6981. Tools are also provided which allow a user to update the database to include new entries as they are added to the PDB and/or their own proprietary structures.

In cases where there is no template available for a loop, the prediction should be viewed with scepticism. It may be possible to improve the prediction method to reduce the impact of missing templates in future.

The method is quick, as it is generally not necessary to build multiple structures or perform long refinement cycles to generate good models. However, the analysis of template choices suggests that loop modeling may sometimes benefit from the use of more than three templates to reduce the impact of a template with an anomalous conformation, and that it could be useful to generate multiple loop models and discard those belonging to minor clusters.

While the use of canonical filtering will in many cases choose appropriate templates, this needs to be balanced against other factors in particular the sequence similarity of the loop itself, as shown in the case of 4JG1. Some of the canonical definitions including kL1:2A and kL2:1 may be overly restrictive in the specificity of residues required at certain positions; these should be reviewed

It has been found that in addition to the similarity of the Fv region, the structure will depend on whether the type of constant light chain is lambda or kappa [28]. A possible improvement to the method would be to check that all the framework templates derived from structures with the appropriate constant domain type.

The positioning of the N-terminal section of the chains may be particularly sensitive to the conformational differences which may be present in the templates due to the commonly-found proline in the L-chain or adjacent glycine residues in the H chain.

Long loops can show considerable variation for different chains within the same crystal structure. It seems likely that, especially for long loops which contain glycine residues, the conformation observed in a particular crystal environment may not necessarily be more ‘correct’ than another. The analysis of other loops indicates that quite subtle changes in the chemistry can lead to different non-bonded interactions and hence loop conformations.

The presence of cis-peptides either in templates when not found in the X-ray structure or vice-versa can lead to discrepancies. While a non-proline cis-peptide arising in a prediction as a result of a cis-proline in a template could perhaps be avoided, it is not clear what other measures could be taken to avoid this.

## Supporting information

S1 Text List of PDB Ids for the validation set. (TXT)

S1 Table Summary RMSD tables. Contains the RMSD values of predicted versus x-ray structure for the various regions, and details of the framework and CDR templates for each target.
(ZIP)

S1 Dataset Predicted structures. Predicted structures using the Top5 framework method, Chothia CDR definition with no canonical filtering and generating 50 models. These were used in the detailed discussion of outliers and for comparison against predictions using canonical filtering.
(ZIP)

S2 Dataset Predicted structures. Predicted structures using the Top5 framework method, Chothia CDR definition with canonical filtering and generating 50 models. These were used in the comparison against predictions using no filtering.
(ZIP)

S3 Dataset Predicted structures. Predicted structures using the Top5 framework method, Chothia CDR definition with canonical filtering, using up to 5 CDR templates rather than the default 3, and generating 10 CDR models.
(ZIP)

S1 File Chothia canonical loop definition (CSV)
